# The Canadian Multi‐Ethnic Research on Aging (CAMERA) Study: Objectives and Design

**DOI:** 10.1002/alz.095035

**Published:** 2025-01-09

**Authors:** Tulip Marawi, Harleen Rai, Alexander J Nyman, Georgia Gopinath, Madeline Wood Alexander, Rachel Yep, Silina Z Boshmaf, Douglas P Munoz, Walter Swardfager, Sandra E Black, Maged Goubran, Jennifer S Rabin

**Affiliations:** ^1^ Sunnybrook Research Institute, Toronto, ON Canada; ^2^ University of Toronto, Toronto, ON Canada; ^3^ Queen’s University, Kingston, ON Canada; ^4^ Department of Pharmacology and Toxicology, University of Toronto, Toronto, ON Canada; ^5^ Division of Neurology, Department of Medicine, University of Toronto, Toronto, ON Canada; ^6^ Department of Medical Biophysics, University of Toronto, Toronto, ON Canada; ^7^ Harquail Centre for Neuromodulation, Sunnybrook Research Institute, Toronto, ON Canada

## Abstract

**Background:**

Despite being among the fastest‐growing ethnoracial groups in Canada and the United States, individuals of Asian descent are significantly underrepresented in research on Alzheimer’s disease and related dementias (ADRD). Limited evidence suggests a lower incidence of dementia among Asian Americans compared to their White counterparts; however, there is variability in risk factors among Asian American subgroups. Understanding this heterogeneity is crucial for tailoring dementia prevention and intervention strategies in Asian populations. To address this gap, we launched the CAnadian Multi‐Ethnic Research on Aging (CAMERA) study in Toronto, Canada.

**Method:**

CAMERA is a 5‐year longitudinal observational study based at Sunnybrook Health Sciences Centre in Toronto, Canada. CAMERA aims to enroll 300 cognitively unimpaired adults aged 55‐85 who self‐identify as South Asian, Chinese, or non‐Hispanic White (n = 100/group). Participants are recruited from the greater Toronto area using culturally appropriate, community‐based participatory approaches. We formed a community advisory board (CAB) comprising community members who share the same identity, language, culture, and values as CAMERA participants. The CAB actively engages in the research process, participant recruitment, research questions, and knowledge translation. In terms of the study protocol, at baseline (Year 1), Year 3, and Year 5, participants complete clinical, cognitive, brain MRI, blood (for genetic and biomarker analysis), and language‐ and culturally neutral eye‐tracking assessments. Every year, participants complete a series of questionnaires via REDCap covering demographic, community, cultural and social, mental and physical health, and sex‐specific health factors.

**Result:**

As of April 2024, we have enrolled 131 participants. Of those, 92 participants completed baseline Year 1 testing. Of the 92 participants, 31 (33.7%) self‐identified as South Asian, 36 (39.1%) as Chinese, and 25 (27.2%) as non‐Hispanic White. Participants have a mean age of 68.1 years (SD = 6.2), and are mostly female (62 (67.4%)).

**Conclusion:**

To our knowledge, this will be the first longitudinal cohort study that brings together sensitive brain imaging tools, novel blood‐based markers, and comprehensive cognitive data to investigate ADRD risk in understudied Asian subgroups. This research will yield currently unavailable data to inform the development of precision strategies to prevent and delay ADRD in Asian and other populations.

